# FGF18 induces chondrogenesis and anti-osteoarthritic effects in a mouse model for TMJ degeneration

**DOI:** 10.1371/journal.pone.0317816

**Published:** 2025-04-24

**Authors:** Flavia Gomes Velasque Gama, Christina Casciani, Eliane Hermes Dutra

**Affiliations:** Department of Orthodontics, UConn Health, Farmington, Connecticut, United States of America; Texas A&M University College of Dentistry, UNITED STATES OF AMERICA

## Abstract

**Objective:**

Temporomandibular Joint Osteoarthritis (TMJ-OA) is a degenerative disease characterized by progressive loss of cartilage and subchondral bone sclerosis. Currently there are no effective treatments for TMJ-OA. FGF18 is a member of the fibroblast growth factor family with essential roles for chondrogenesis, selectively binding to FGFR3 receptor. Studies have reported FGF18 attenuates cartilage degradation. Whereas the anti-osteoarthritic effects of FGF18 in the articular cartilage are known, the effects of FGF18 in a TMJ fibrocartilage degeneration mouse model remain to be determined. The goal of this project was to determine the effects of intra-articular injections of FGF18 in a mouse model for TMJ degeneration.

**Method:**

Prosthesis tubes were bonded at the left lower incisor of 6-week-old triple collagen transgenic mice (Col1a1XCol2a1XCol10a1), creating unilateral crossbite and degeneration of the TMJ fibrocartilage. Six weeks after placement of prosthesis tubes, experimental and control mice received intra-articular injections of rmFGF18 (5µg/week) or saline, respectively, for 3 weeks.

**Results:**

Mice receiving saline intra-articular injections presented with a thinner cartilage layer with decreased proteoglycan distribution and Edu positive cells (chondrocyte proliferation marker), while mice injected with rmFGF18 presented with significant increased fibrocartilage thickness, remarkable proteoglycan distribution and chondrocyte proliferation, suggesting healing of the induced degeneration. Furthermore, reversal of the TMJ degeneration achieved by rmFGF18 injection was accompanied by a substantial reduction in Noggin (antagonist of BMP signaling), increase in TIMP1 (inhibitor of metalloproteinases such as MMP13) and decrease in MMP13 expression.

**Conclusion:**

Our results postulate FGF18 as a powerful growth factor for the healing of TMJ fibrocartilage.

## Introduction

Osteoarthritis (OA), or degenerative arthritis, is the most common degenerative disease of the joints. It is a disease of the whole joint and not only the cartilage, characterized by progressive loss of articular cartilage, formation of subchondral bone sclerosis and osteophytes. The clinical signs of the disease are joint pain with activity, reduced range motion, crepitus and swelling. Currently, treatments for OA are limited to educate the patient about the disease and its management, to control pain and improve joint function with medications [1].There is no available treatment to cure OA; loss of the articular cartilage is progressive and irreversible. OA can involve the temporomandibular joint (TMJ) as well [2]. TMJ-OA significantly impairs patients’ quality of life by causing acute and chronic pain. The available treatments for TMJ-OA include non-surgical approaches, such as occlusal appliances, cold and warm packs, medications and physiotherapy, and surgical options such as arthrocentesis or joint replacement. These treatments only control patients’ symptoms and do not cure TMJ-OA [3].

The Fibroblast Growth Factor (FGF) family is comprised of 22 FGF genes arranged into seven subfamilies containing two to four members each. FGF18 is a member of the Fgf8 subfamily, with essential roles for endochondral ossification and chondrogenesis [4–7]. The FGF Receptor (Fgfr) gene family is comprised of four members, Fgfr1-Fgfr4 tyrosine kinases. FGF18 selectively binds to FGFR3 receptor [7,8]. Our preliminary data have shown that FGF18 protein expression and phosphorylation of FGFR3 are significantly reduced in the cartilage of the TMJ as mice age, suggesting that lack of FGF18 signaling could contribute to the development of TMJ-OA [9]. Additionally, studies have shown that intra-articular injections of FGF18 may attenuate articular cartilage degradation [10–13]. Previous reports have shown enhanced chondrocyte proliferation, cartilage thickness, attenuation of cartilage degradation, increase in collagen II deposition and suppression of matrix metallopeptidase 13 (marker for cartilage degeneration), suggesting reversal of induced injury in the articular cartilage of rodents by FGF18 [11,12]. In addition, the effects of Recombinant Human FGF18 (rhFGF18, Sprifermin) were studied *in vitro* using porcine and human chondrocytes. RhFGF18 stimulated cell growth and increased the number of matrix-producing chondrocytes [14]. Sprifermin was also tested in a clinical trial for the treatment of osteoarthritis of the knee (The FORWARD (FGF-18 Osteoarthritis Randomized Trial with Administration of Repeated Doses) [15–17], in which suggested that repeated doses of Sprifermin increases cartilage thickness, and reduces cartilage loss over 3.5 years post-treatment. Moreover, a systemic review evaluated the effects of rhFGF18 for articular cartilage healing in preclinical controlled trials, concluding that rhFGF18 improves cartilage defect repair [13]. Recently, increased FGF18 expression has been found in patients with femoroacetabular impingement (FAI) [18], suggesting a chondroprotective role of FGF18 in the femoral head articular cartilage. Although the mentioned results suggest promising protective and healing effects for TMJ-OA, articular cartilage and fibrocartilage of the TMJ have distinct cellular and molecular features and may respond differently to injury and therapeutical interventions [19]. Whereas the anti-osteoarthritic effects of FGF18 in the articular cartilage are known [10–14,20], the effects of FGF18 in a TMJ fibrocartilage degeneration mouse model remain to be determined.

The goal of this project was to determine the effects of an intra-articular injection of FGF18 in a mouse model for TMJ degeneration. We hypothesize that intra-articular administration of Recombinant Mouse FGF18 (rmFGF18) will increase the number of chondroprogenitors and will aid in the repair and regeneration of the fibrocartilage.

## Method

### Ethics statement

Experiments were approved (protocol number AP-200736–0925) by the Institutional Animal Care and Use Committee (IACUC) at the University of Connecticut Health. Animals were anesthetized by a ketamine/xylazine cocktail before bonding the tubes and before the TMJ intra-articular injections. Animals were euthanized by carbon dioxide (CO2) inhalation at the end of experiments.

### Experimental design

Triple collagen transgenic mice (Col1a1xCol2a1xCol10a1) were used for this study. This model contains fluorescent reporter expression with the 3.6-kb fragment of the rat collagen type I promoter fused to a topaz-fluorescent protein (Col3.6-tpz), collagen type II promoter fused to a cyan-fluorescent protein (Col2-cyan), and collagen type X promoter fused to cherry-fluorescent protein (Col10-cherry), in a CD-1 background. There is a Col3.6-tpz to Col2-cyan and ultimately to Col10-cherry cell maturation lineage in the cartilage of the TMJ. Therefore, Col3.6-tpz (Col1a1, Col type I) represents undifferentiated polymorphic/flattened mesenchymal-like cells; Col2-cyan (Col2a1, Col type II) is a marker for flattened/pre-hypertrophic chondrocytes, while Col10-cherry (Col10a1, Col type X) is expressed in the deeper layers of the cartilage, containing hypertrophic chondrocytes [21].

Thirty-two 6-week-old Col1a1xCol2a1xCol10a1 male mice were divided in 4 groups:

Control Saline: no TMJ degeneration + saline injections (N=8).

Control FGF18: no TMJ degeneration + rmFGF18 injections (N=8).

Degeneration Saline: TMJ degeneration + saline injections (N=8).

Degeneration FGF18: TMJ degeneration + rmFGF18 injections (N=8).

We powered our study using preliminary data (parameter used: cartilage thickness and OARSI score). To detect the difference between groups, using a two-sided two- sample t-test, we determined 8 animals per group were needed.

### TMJ fibrocartilage degeneration model

**Fig 1:**
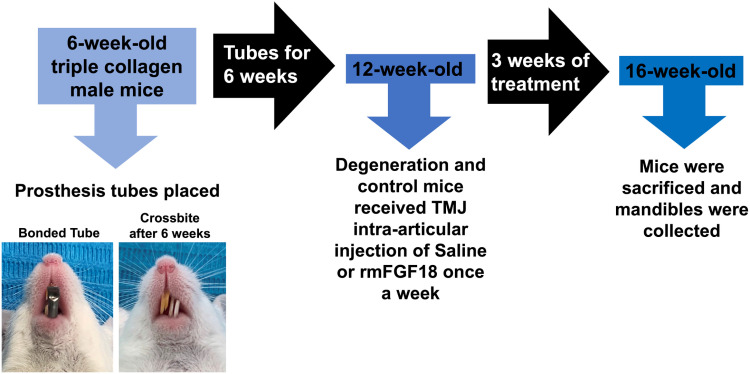
Experimental TMJ degeneration. Prosthesis tubes were bonded unilaterally on one incisor of six-week-old mice. Tubes were maintained for 6 weeks when treatment of experimental and control mice started.

Degeneration of the fibrocartilage of the TMJ was induced in *Degeneration Saline* and *Degeneration FGF18* groups by bonding a metal tube on one incisor to create a unilateral crossbite (**Fig 1**). The TMJ degradation model has been stablished before [22]. Metal tubes were made of a pinhead of 0.90 mm telescopic tube (Morelli, Sorocaba, SP, Brazil). Tubes were curved to form a 135° labially inclined occlusal plane.

Mice were first anesthetized with ketamine/xylazine. The surface of the incisors was treated with a self-etching primer (3M™ Transbond™ Plus Self Etching Primer, 3M, St. Paul, MN, USA) and tubes were bonded using orthodontic adhesive (Transbond XT, 3M, St. Paul, MN, USA). Metal tubes were bonded on the mandibular left incisors for both TMJ degradation groups.

Mice received crushed regular mouse diet for 2 weeks after the placement of tubes to allow proper feeding. Tubes were maintained for 6 weeks to induce degeneration of the TMJ cartilage. Mice were examined three times per week for stability of tubes and weight changes. Mice losing 20% or more of its weight in 24 hours 136would have been excluded from study. All mice developed a unilateral crossbite towards the left side during this period (**Fig 1**). *Control* mice received no metal tubes.

### TMJ intra-articular injections

Six weeks after the placement of metal tubes, control and degeneration groups received TMJ intra-articular injections of saline or rmFGF18 (Recombinant Mouse FGF18, 5µg/week; NOVUS Biologicals, LLC, Centennial CO, USA) once a week for 3 weeks. The intra-articular injections were performed by the same operator (FGVG – blind for the group allocation), at the right side TMJ for all animals. This side was chosen based on a pilot study in which we observed that unilateral crossbite towards the left side induces degeneration at the right TMJ.

Mice were anesthetized with a ketamine/xylazine cocktail before injections. The periauricular area was then cleaned with isopropyl alcohol. An imaginary line was traced from the level of eye to the ear canal. The injection site was at the end of zygomatic arch (located by palpation), at the imaginary line between eye and ear canal. The location was confirmed by pressing the periauricular region with a tip of college plier and checking the movement of the mandible. Once the location was confirmed, the insulin needle was inserted under the zygomatic arch. The needle should be inserted about 1.5–2mm, when a bony structure can be felt. A total volume of 30µl containing 5µg of rmFGF18 or saline was used.

In addition, mice received intraperitoneally injections of Edu (5-ethynyl 2´-deoxyuridine, Click-IT Edu cell proliferation assay, Invitrogen, Waltham, MA, USA) two days and one day before euthanasia for chondrocyte proliferation analysis.

Mice were euthanized one week after the last weekly treatment of TMJ intra-articular injections (Saline or rmFGF18).

### Histological analysis

Mandibular condyles were dissected and fixed for 48 hours in 10% formalin then placed in 30% sucrose overnight. Samples were then embedded in cryomedium (Thermo Shandon, Pittsburgh, PA, USA) using disposable molds (Thermo Shandon, Pittsburgh, PA, USA). The medial surfaces of the samples were embedded against the base of the mold, parallel to the floor of the mold. Specimens were stored at −20°C before sectioning. Frozen histological sections (5μm thickness) were performed using a Leica Cryostat (Nussloch, Germany). Sections were transferred to slides using the tape transfer method [23].

Sections were first scanned for Col type I (Col3.6-tpz), Col type II (Col2-cyan) and Col type X (Col10-cherry). Baseline imaging of sections for the three channels was performed using the observer ZI fluorescent microscope (Carl Zeiss, Thornwood, NY, USA). Next, the coverslip was removed by soaking slides in PBS and sections were then stained for Edu (Click-IT Edu assay, Invitrogen, Waltham, MA, USA) and DAPI (4’,6-diamidino-2-phenylindole), generating a yellow fluorescent signal for proliferating chondrocytes, counterstained with blue DAPI signal for nuclear staining. After imaging for Edu, the coverslip was removed, and the same slide was stained for Toluidine Blue and Safranin O.

Additional slides were used for immunofluorescence of TIMP1, MMP13 and NOGGIN (antibodies from ABCAM, Cambridge, MA, USA), also counterstained with DAPI.

### Quantification of images

Histological sections were quantified using the ImageJ software (National Institutes of Health, Bethesda, MD, USA). Three sections of each individual sample were quantified. Cartilage thickness was measured in Toluidine Blue stained sections, measured at 3 different points on each image. Proteoglycan area and OARSI semi-quantitative score [24] were determined in Safranin O stained images.

Col type I, Col type II and Col type X positive cells were quantified by counting the number of positive pixels (green, blue, and red, respectively) and dividing by the total number of pixels in the cartilage area. Similarly, Edu, TIMP1, MMP13 and NOGGIN expressions were also quantified by measuring the number of stained pixels over the total number of pixels in the cartilage (Yellow for Edu, MMP13 and NOGGIN and green for TIMP1).

### Statistical analysis

Three sections per sample (N=8) were analyzed and quantified. Statistical comparison between groups were determined by Ordinary one-way analysis of variance (ANOVA) and Tukey’s post-hoc multiple comparisons. Statistical tests were two sided and a *p* value of < 0.05 was considered statistically significant. Statistical analysis was performed using Graph Pad Prism (San Diego, CA, USA).

## Results

All mice included in the study presented with no systemic alterations observed by gross visual inspection and weight monitoring. Mice subjected to experimental TMJ degeneration lost some weight the first week after the tubes were bonded, but no mouse lost more than 10% of its weight per day. Mice continue to gain weight after the second week of the experimental phase. There was no statistical significance difference in the final weight for the Control Saline (40.70 ± 1.442 grams), Control FGF18 (36.23 ± 0.850 grams) and Degeneration FGF18 (36.38 ± 1.607 grams) groups, however the Degeneration Saline group presented with a significant lower final weight (30.53 ± 3.347 grams) in relation to all other groups (S1 Fig).

All histological analysis were performed on the right TMJ, except for the cartilage thickness, in which both sides were analyzed to confirm the contralateral side was not affected.

**Fig 2:**
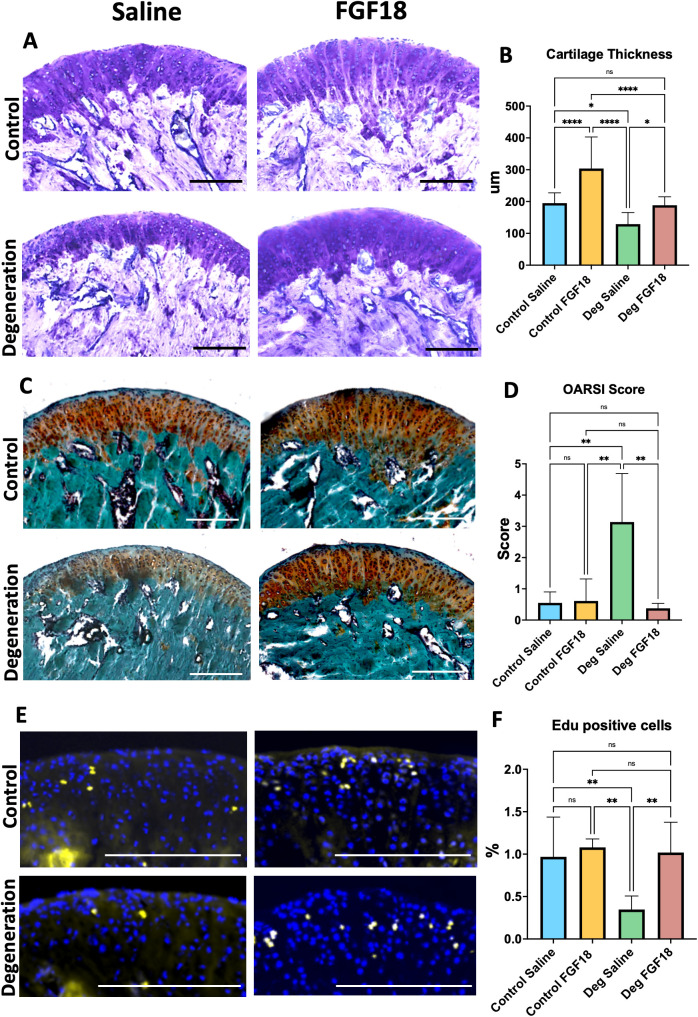
FGF18 injections increase cartilage thickness, chondrocyte proliferation and induce healing after TMJ experimental degeneration. Sagittal sections of stained for Toluidine Blue (A), Safranin **O** (C) and Edu (E). Scale bar = 200µm. Histograms (B, D and F) represent mean, standard deviation, and statistical significance by ANOVA between groups. N= 5 per group. Ns = non-significant; ** **p** = 0.001; *** **p** = 0.0001; **** **p** < 0.0001.

Mice subjected to TMJ degeneration receiving saline intra-articular injections presented with a thinner cartilage thickness in comparison to controls (Saline and FGF18) and to FGF18 Degeneration groups (**Fig 2A and 2B**). Similarly, the Saline Degeneration group showed decreased proteoglycan distribution and increased OARSI semi-quantitative score^7^ in comparison to FGF18 Degeneration and controls groups (**Fig 2C and 2D**). There was no statistical difference for cartilage thickness in the contralateral side (left TMJ) of all groups (S2 Fig), suggesting the contralateral side was not affected by the injections performed in the right side or induction of unilateral crossbite.

**Fig 3:**
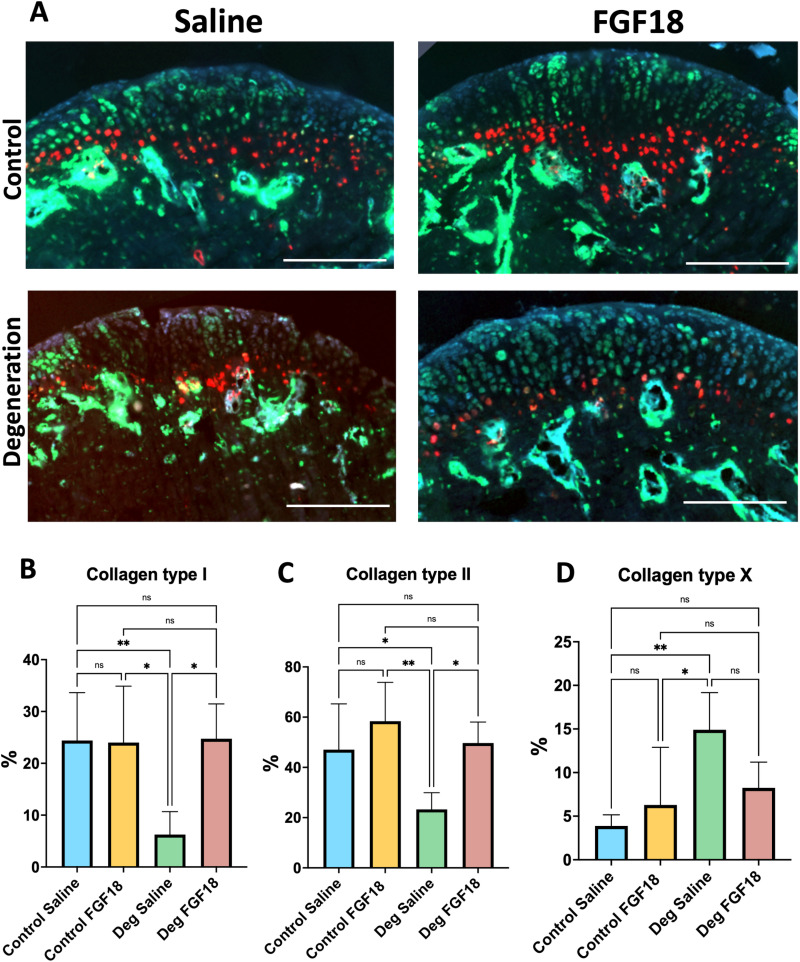
FGF18 injections increase chondroprogenitors and extracellular matrix and reduce chondrocyte hypertrophy after TMJ experimental degeneration. Fluorescent reporters for Col I (Col1a1, green), Col **II** (Col2a1, blue) and Col X (Col10a1, red). Scale bar = 200µm. Histograms (B, C and D) represent mean, standard deviation, and statistical significance by ANOVA between groups. N= 5 per group. Ns = non-significant; * **p** < 0.05; ** **p** = 0.001.

Saline Degeneration mice presented with decreased Edu positive cells (chondrocyte proliferation marker), while the Degeneration group injected with FGF18 presented with a percentage of proliferative chondrocytes comparable to control groups (**Fig 2E and 2F**). Moreover, a significant decrease in Col I cells (a marker for chondrocyte proliferation and chondroprogenitors) and Col II cells (a marker for extracellular matrix) in the TMJ fibrocartilage of Degeneration Saline mice in comparison to Degeneration FGF18 and Control groups was noticed (**Fig 3A**, **3B and 3C**). Next, we analyzed the distribution Col X (a marker for hypertrophic chondrocytes and degeneration) and noticed an increase on this type of cell in Degeneration Saline mice in comparison to Degeneration FGF18 and Control groups (Fig 3A **and** 3D**).** We also observed an invasion of Col X cells into the cartilage in the Degeneration Saline group - an effect that seems to be reversed in Degeneration FGF18 group (Fig 3A).

**Fig 4:**
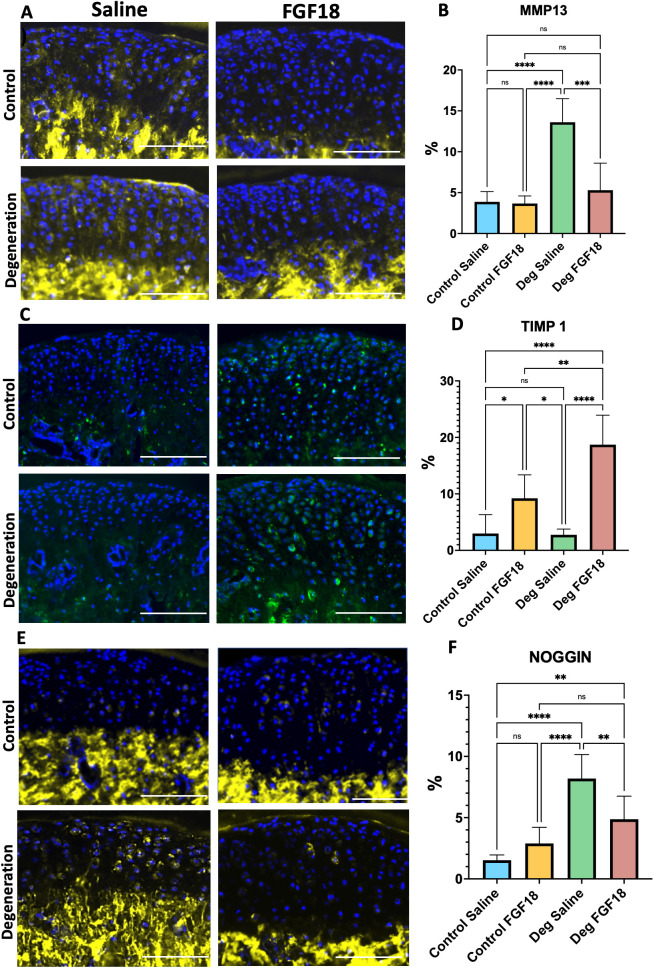
FGF18 injections increase TIMP1 and decrease MMP13 and NOGGIN, suggesting healing of degeneration induction of anabolic effect. Immunofluorescence for MMMP13 (A, MMP13 yellow signal counterstained with blue DAPI), TIMP1 (C, TIMP1 green signal counterstained with blue DAPI) and NOGGIN (E, NOGGIN yellow signal counterstained with blue DAPI). Scale bar = 100µm. Histograms (B, D and F) represent mean, standard deviation, and statistical significance by ANOVA between groups. N= 5 per group. Ns = non-significant; * **p** < 0.05; ** **p** = 0.001; *** **p** = 0.0001; **** **p** < 0.0001.

Furthermore, reversal of the TMJ degeneration achieved by FGF18 injections was accompanied by a substantial decrease in MMP13 (Fig 4A **and** 4B), increase in TIMP1 (Fig 4C **and** 4D) as well as a reduction in Noggin expression (Fig 4E **and** 4F).

## Discussion

Our data shows, for the first time, that administration of intra-articular injections of FGF18 into the TMJ of mice with induced cartilage degeneration led to an increase in cartilage thickness, proteoglycan distribution, proliferation of chondroprogenitors and decreased markers for osteoarthritis, suggesting that FGF18 promotes repair of the TMJ fibrocartilage.

The TMJ intra-articular injections (FGF18 or saline) were initiated 6 weeks after tubes were bonded on mice, when unilateral crossbite and degeneration of TMJ cartilage could be detected. The thickness of the TMJ cartilage was significantly reduced in the Degeneration Saline group when compared to mice without cartilage degeneration receiving saline injections only (Control Saline group). In contrast, the 3-week treatment with FGF18 injections in mice with degeneration of the TMJ cartilage showed that intra-articular injections of FGF18 repaired the cartilage thickness loss (Degeneration FGF18 group). These results are consistent with previous studies in rats with a surgical meniscal tear submitted to a FGF18 treatment. Injections of FGF18 in the meniscus of rats induced a dose-dependent increase in cartilage thickness of the articular surface of the medial tibial plateau, while cartilage thickness decreased with time for rats receiving no treatment after the surgical procedure [11]. Similarly, it has been demonstrated that injections of AAV2-FGF18 or rhFGF18 increase cartilage thickness of the anterior horn of the medial meniscus in rats [25]. The FORWARD trial assessed efficacy and safety of intra-articular injection of sprifermin in patients with knee osteoarthritis. Increase in articular cartilage thickness, as well as long-term structural cartilage modification after 3.5 years of Sprifermin treatment have been reported [15–17].

Proteoglycans, which consist of a protein core with glycosaminoglycans (GASGs) attached, are found within the collagen network of cartilage, and play a key role in resisting compressive forces. Aggrecan is the major proteoglycan present in the condylar mandibular cartilage and is fundamental for the structural and functional integrity of the TMJ cartilage [26]. In our study, the proteoglycan distribution and pathological score for OA (OARSI) [24] was determined by Safranin-O staining. The OARSI score system quantifies the severity of cartilage damage, evaluating the cartilage proteoglycan distribution and the architecture of the cartilage with grades from 0 to 6. The higher the score, the lower the proteoglycan distribution, and the less desirable cartilage structure. In this experiment, the highest scores were attributed to the TMJ cartilage of the Degeneration Saline group, which showed proteoglycan loss as the intensity of the staining decreased with the defective cartilage structure. The Degeneration FGF18 group received the lowest scores, due to the robust proteoglycan distribution and the best cartilage structure. The Safranin-O-stained sections suggest the cartilage was repaired by FGF18 treatment (Degeneration FGF18 group). Comparable results were shown by an experiment performed in rats with induced post-traumatic osteoarthritis (PTOA) after transecting the anterior cruciate ligament and medial meniscus destabilization treated with intra-articular injections of Recombinant Rat FGF18 Protein twice a week. The PTOA group presented with significant damage of the cartilage and loss of proteoglycan and glycosaminoglycan when no FGF18 treatment was performed. Conversely, the group with PTOA treated with FGF18 significantly reversed OA changes and the OARSI scores decreased significantly compared with the group without FGF18 injections [12].

The mandibular condylar cartilage is essential for TMJ function, enhancing the articulation within the TMJ and lessening loads to the subchondral bone [27]. Mandibular condylar cartilage is a type of fibrocartilage composed of different zones: articular zone of fibrous connective tissue, proliferative zone of undifferentiated mesenchymal cells, cartilage mature and hypertrophic zones [28,29]. Mandibular condylar cartilage has type I collagen mostly in the superficial zones, while collagen type II and X are dominant in the mature and hypertrophic zones, respectively [30,31].

Collagen type II has been long regarded as the main component of the cartilage matrix but has also been linked to suppression of chondrocyte hypertrophy and OA progression [32]. It has been shown that FGF18 treatment remarkedly increased collagen type II expression in surgically induced OA in the knees of rats [12]. Our results corroborate with the mentioned experiment; we found an increase in collagen type II after FGF18 treatment of the degenerated TMJ cartilage. Furthermore, collagen type I has also increased as a result of FGF18 treatment (Degeneration FGF18 group). Cells expressing collagen type I represent the fibrocartilage layer of the TMJ cartilage. It is the outer layer of the cartilage, containing mesenchymal-like cells that can differentiate into collagen type X cells [21]. An increase in collagen type I cells means an increase in chondrocyte progenitors (as validated by an increase in EdU positive cells), indicating an anabolic effect in the cartilage of the TMJ.

Type X collagen, expressed by hypertrophic chondrocytes, is a marker for OA [33]. In the present study, type X collagen cells invaded the fibrocartilage of the Degeneration Saline group, suggesting the eventual replacement of cartilage by the underlying subchondral bone. A different finding was observed on the Degenerative FGF18 group, where type X collagen cells did not seem to invade the cartilage, showing comparable distribution of hypertrophic chondrocytes to the Control groups. The combined changes in collagen expression (increase in type I and II and decrease in type X collagen) suggest that FGF18 treatment was able to reverse the pathological changes (degeneration) created by the unilateral crossbite.

TMJ-OA is associated with abrasion and deterioration of the cartilage. Cartilage degradation is mediated by collagenases such as the matrix metalloproteinases (MMPs) and proteases such as aggrecanases [2]. MMP13 is a major marker for OA, targeting degradation of type II collagen and proteoglycans [34,35]. In the present study, the Degenerative Saline group showed increased MMP13 expression (as expected), while the Degenerative FGF18 group presented a reduction of this degeneration marker. Similar results were obtained by Yao et al [12], who found that FGF18 treatment suppressed the expression of MMP13, showing that intra-articular injections of FGF18 have anti-osteoarthritic effects on rats undergone to previous knee surgery [12]. TIMP1 (Tissue Inhibitor of Metalloproteinases) is a glycoprotein serving as a natural inhibitor of matrix metalloproteinase, including MMP13 [36], and has been considered a target of FGF18 for its anticatabolic effects in the articular cartilage [10]. We observed an increase in TIMP1 in both groups that received TMJ intra-articular injections (Control FGF18 and Degeneration FGF18). Taking these observations together, we can postulate that the regenerative effects of intra-articular injections of FGF18 in the TMJ are mediated by an increase in TIMP1 and a subsequent decrease in MMP13.

Noggin is another target of FGF18 during chondrogenesis [7,37]. Noggin is a secreted polypeptide expressed in all developing cartilages which binds and inactivates Bone Morphogenetic Proteins (BMP) 2, 4, 6 and 7 [38]. It has been suggested that FGF18 does not directly induce gene expression of BMP signaling, but rather suppresses the expression of noggin, BMP antagonist, to facilitate chondrogenesis [37]. Our results show significant increase in noggin expression in Degeneration Control group, while mice induced to TMJ degeneration treated with intra-articular injections of FGF18 showed a substantial decrease in noggin in relation to the former group, suggesting that FGF18 might be indirectly inducing BMP signaling for the anabolic effects in the TMJ fibrocartilage.

One of the limitations of this study is the inclusion of male mice only for our initial analysis. We plan to add female groups of mice to confirm the effects observed in male mice. Our future directions also include to test if TMJ intra-articular injections of FGF18 could prevent degeneration.

These results suggest that TMJ intra-articular injections of FGF18 increase cartilage thickness, proteoglycan distribution, proliferation of chondroprogenitors and decrease markers for osteoarthritis in the fibrocartilage of TMJ after experimental degeneration, postulating FGF18 as a powerful growth factor for the healing of TMJ fibrocartilage.

## Supporting Information

S1 Fig Degeneration Saline group presented with significant decreased final weight in relation to other groups. N= 5 per group. Ns = non-significant; * p < 0.05; *** p = 0.0001.
(TIF)

S2 Fig Equal cartilage thickness for all groups, suggesting the contraleteral (left side) was not affected by right side injections or unilateral crossbite. N= 5 per group. Ns = non-significant.
(TIF)
