# Supplementary figures and images for: FGF18 induces chondrogenesis and anti-osteoarthritic effects in a mouse model for TMJ degeneration
Source: PLoS One. 2025 Apr 24;20(4):e0317816. doi: 10.1371/journal.pone.0317816 (PMC12021239; doi:10.1371/journal.pone.0317816)

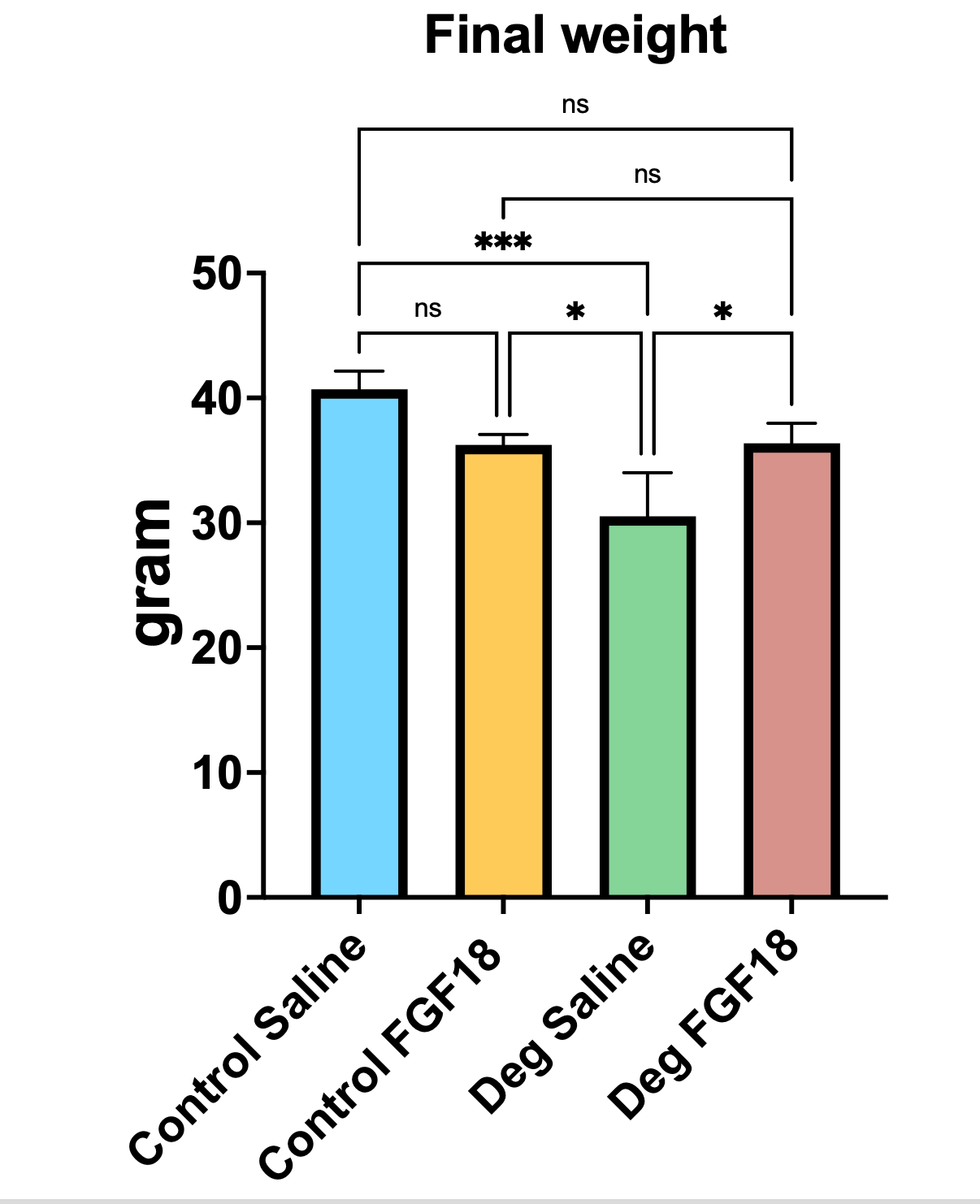

Supplement: S1 Fig — N= 5 per group. Ns = non-significant; * p < 0.05; *** p = 0.0001. (TIF) [file pone.0317816.s001.tif]

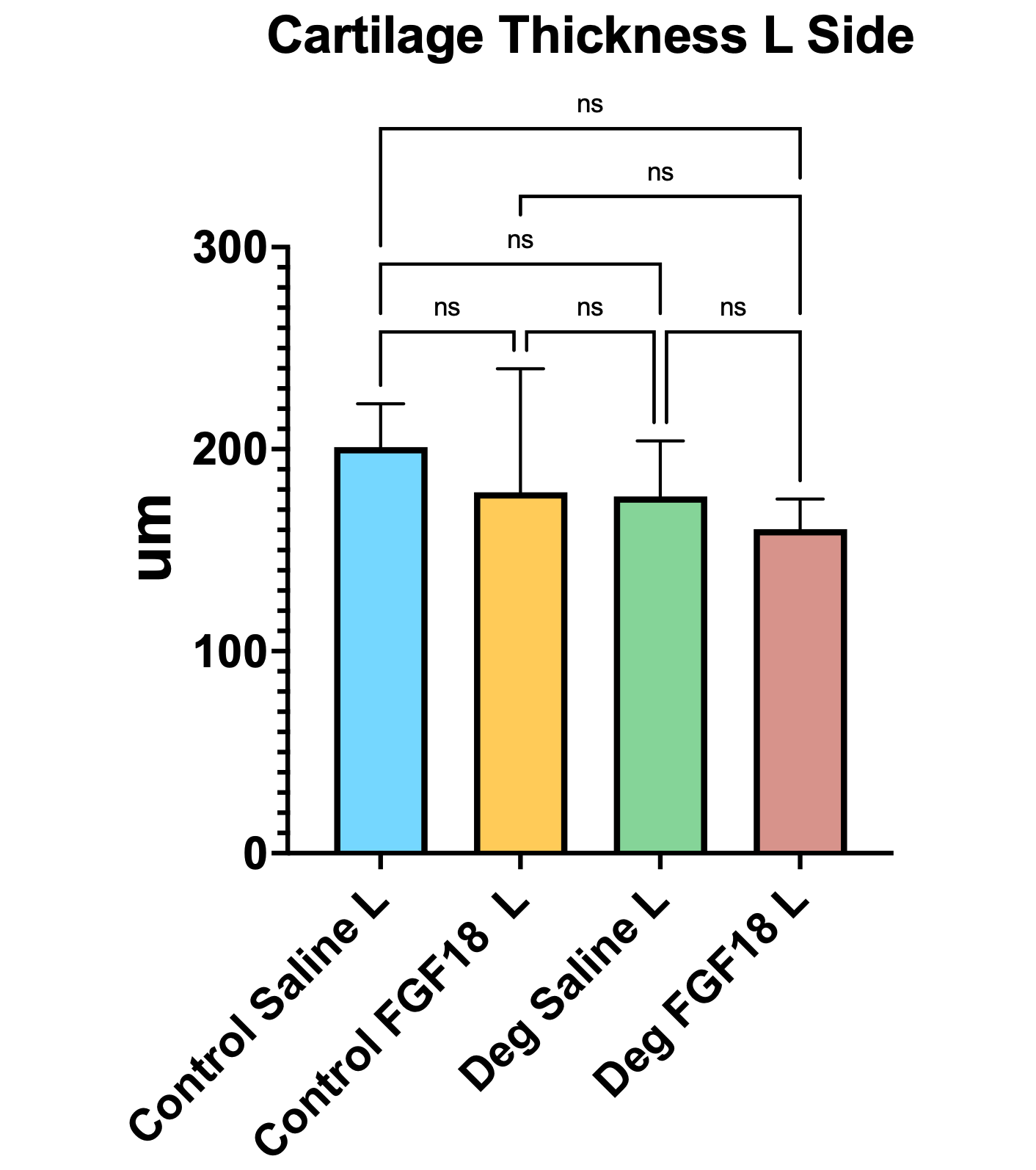

Supplement: S2 Fig — N= 5 per group. Ns = non-significant. (TIF) [file pone.0317816.s002.tif]
